# Ipsilateral common iliac artery plus femoral artery clamping for inducing sciatic nerve ischemia/reperfusion injury in rats: a reliable and simple method

**DOI:** 10.1186/1749-7221-3-27

**Published:** 2008-12-22

**Authors:** Mohsen Nouri, Reza Rahimian, Gohar Fakhfouri, Mohammad R Rasouli, Sanaz Mohammadi-Rick, Anita Barzegar-Fallah, Fahimeh Asadi-Amoli, Ahmad Reza Dehpour

**Affiliations:** 1 Department of pharmacology, Tehran University of Medical Sciences, Tehran, Iran; 2 Department of pharmacology, Shahid Beheshti University of Medical Science, Tehran, Iran; 3 Department of pathology, Tehran University of Medical Sciences, Tehran, Iran

## Abstract

The aim of this study was to develop a practical model of sciatic ischemia reperfusion (I/R) injury producing serious neurologic deficits and being technically feasible compared with the current time consuming or ineffective models. Thirty rats were divided into 6 groups (n = 5). Animal were anesthetized by using ketamine (50 mg/kg) and xylazine (4 mg/kg). Experimental groups included a sham-operated group and five I/R groups with different reperfusion time intervals (0 h, 3 h, 1 d, 4 d, 7 d). In I/R groups, the right common iliac artery and the right femoral artery were clamped for 3 hrs. Sham-operated animals underwent only laparotomy without induction of ischemia. Just before euthanasia, behavioral scores (based on gait, grasp, paw position, and pinch sensitivity) were obtained and then sciatic nerves were removed for light-microscopy studies (for ischemic fiber degeneration (IFD) and edema). Behavioral score deteriorated among the ischemic groups compared with the control group (p < 0.01), with maximal behavioral deficit occurring at 4 days of reperfusion. Axonal swelling and IFD were found to happen only after 4 and 7 days, respectively. Our observations led to an easy-to-use but strong enough method for inducing and studying I/R injury in peripheral nerves.

## Background

Ischemia is the subject of investigation in many experimental studies on neuropathies representing neural changes in diabetes mellitus, vasculitis or vasculopathies, vascular occlusion by emboli or thrombosis, and trauma. Histological changes including endoneurial edema, demyelination, axonal degeneration, and diffuse loss of nerve fibers as well as behavioral and electro-physiologic studies are the commonly used measures by researchers to evaluate the outcome of ischemia/reperfusion (I/R) injuries. Setting up a model that is easily performable and induces expected para-clinical and clinical changes in the target organ, is a prerequisite to simulate clinical conditions under laboratory circumstances.

Various models of unilateral I/R injury of rat sciatic nerve have been developed. In one method described by Mitsui et al. [1], the abdominal aorta, the right iliac and femoral arteries, and all identifiable collateral vessels supplying the right sciatic-tibial nerve are ligated for 3 hrs and then reperfused for different time intervals. In another model introduced by Saray and his colleagues [2], only femoral artery and vein – just distal to the inguinal ligament – are clamped for 3 hrs followed by reperfusion. The former is time consuming and requires advanced surgical techniques and instruments and the latter, albeit easily performable, does not produce severe injury [3]. The aim of this study was to develop a practical model producing serious neurologic deficits and still technically feasible. For this purpose, clamping of both the femoral artery and ipsilateral common iliac artery was performed to induce sciatic nerve I/R.

## Materials and methods

Thirty Spraque-Dawley male rats were housed in a temperature-controlled room (25 ± 1°C) and maintained on a 12 h light/dark cycle with free access to food and water. All experiments were performed in accordance with institutional guidelines for animal care and use and also "Principles of laboratory animal care" were followed. The animals weighing 150–200 g were randomly divided into six groups: one control group and five I/R groups at different time intervals of reperfusions (0 h, 3 h, 1 d, 4 d, 7 d).

All animals were anesthetized with ketamine (50 mg/kg) and xylazine (4 mg/kg) and subjected to laparotomy. In the I/R groups, the right common iliac artery and the right femoral artery – just distal to the inguinal ligament – were clamped for 3 hrs using two Yasargil aneurysm clips providing 125 g (1.24 N) force. Reperfusion was checked under a microscope at the distal site of clamping after removing the clips. Using a rectal probe inserted 5 cm into the rectum, the deep rectal temperature was monitored and maintained at 36.5 ± 1°C by a thermal pad. All procedures were carried out for the sham-operated group except for arterial clamping. After certain time intervals of reperfusion, the function of the hind limb was assessed for each animal in terms of behavioral score based on gait, grasp, paw position, and pinch sensitivity, while the score for each index varied from 0 (no function) to 3.0 (normal function) except for pinch sensitivity ranging from 0 to 2 [4]. Behavioral scoring was performed by two separate observers blinded to the status of the rats and the average scores were recorded. Then, the sciatic nerve was fixed in situ for 30 min using 4% formaldehyde in phosphate buffer (pH 7.4) and then, trifurcation of the sciatic nerve was removed, embedded in paraffin, and finally, sections were stained with hematoxylin, eosin and tri-chrome gomori for light-microscopy studies, and were graded for ischemic fiber degeneration (IFD) and edema [5]. The sections were graded 0 to 4 for IFD based on the percentage of IFD as follows: ≤ 2%, 3–25%, 26–50%, 51–75%, and >75%, respectively (Table 1). Three sections in row from each specimen and three random fields at high power field (HPF) level from each section were chosen and the results were averaged. Grading for edema was based on severity and distribution, where 0 = normal, 1 = mild edema, 2 = moderated edema, 3 = severe edema, and 4 = severe and widespread edema (Table 1).

**Table 1:** Histopathological scoring system for quantification of edema and Ischemia Fiber Degeneration (IFD)

Histopathologic grade	Edema	IFD (ischemic fiber degeneration)
0	normal	≤ 2%
1	Mild edema	3–25%
2	Moderate edema	26–50%
3	Severe edema	51–75%
4	Severe and widespread edema	>75%

Non-parametric Kruskal-Wallis (KW) and Mann-Whitney U (MWU) tests were used to compare behavioral and pathologic scores (i.e. edema and IFD grades) among the groups. Data throughout the manuscript are presented as median. All statistical analyses were performed utilizing the SPSS software, version 13.0.

## Results

**Figure 1:**
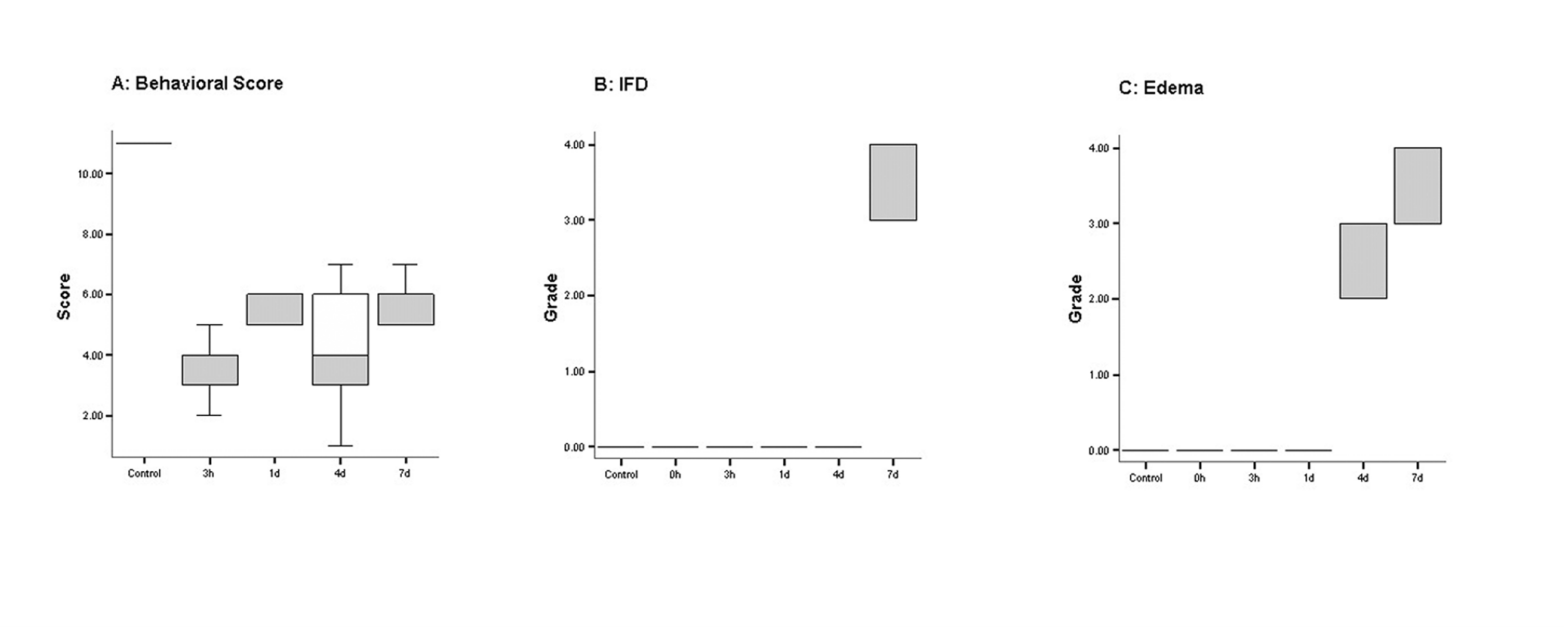
**Behavioral score, the grade of IFD, and edema**. (A) Behavioral score worsens as time passes with the worst outcome at 4 days. (B) IFD occurred only after 7 days of reperfusion while (C) edema happened earlier at 4 days, but was still more severe at 7 days.

**Figure 2:**
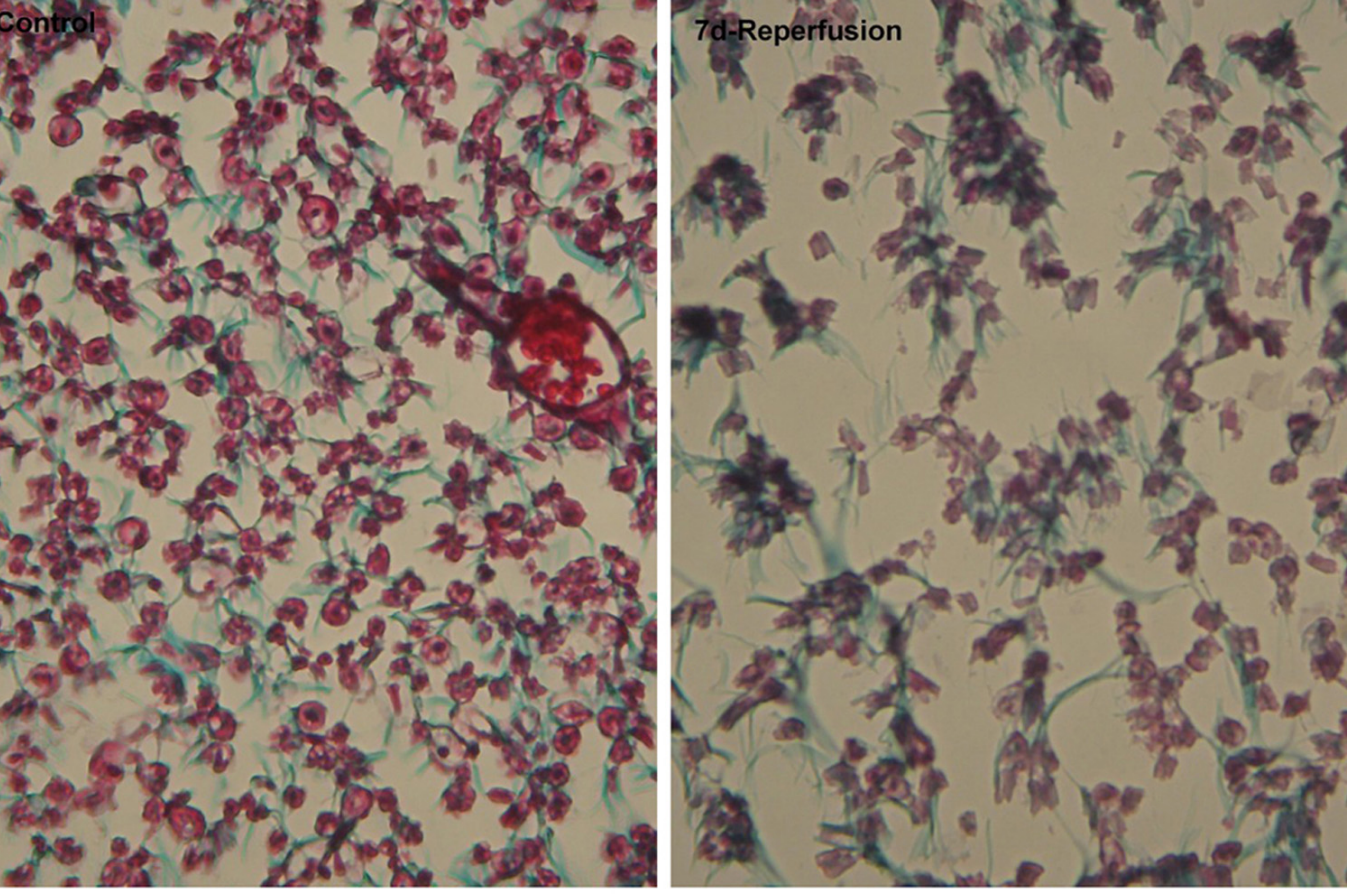
**Slides stained with tri-chrome gomori from control group (left panel) and group 7 days reperfusion (right panel) at light microscopy level**. There were axonal swelling, and fiber degeneration at 7 days compared with control group.

Using KW test, behavioral, IFD, and edema scores of I/R groups differed significantly from those of control (p < 0.01, p < 0.001, and p < 0.001 respectively). Behavioral assessment was not reliable in 0 h reperfused group, because the animals were still under the effect of anesthetic drugs which would interfere with the behavioral outcome. Behavioral function was normal in control group (total score = 11) at any given time. Loss of function was observed in all I/R groups compared with control (group 3 h: score 3.00; group 1 d: score 6.00; group 4 d: score 4.00; group 7 d: score 6.00; MWU, p < 0.01 vs. control) (Fig. 1A). Animals at 4 days showed the worst behavioral outcome, though this was not statistically significant (MWU, p > 0.05). Pathologic changes were significant in the reperfused groups compared with control group (Fig. 2). Fiber degeneration was observed only in group 7 d (group 7 d; 3.00; MWU, p < 0.01) (Fig. 1B). Epineural edema was observed from day 1 on, but marked endoneural edema occurred at 4 (group 4 d; 2.00; MWU, p < 0.01) and 7 days (group 7 d; 4.00; MWU, p < 0.01) (Fig. 1C). Specimens from group 7 d had more severe edema compared with group 4 d (MWU, p < 0.05).

## Discussion

The results of our experiment promised of a newly introduced method for induction of I/R injury in peripheral nerve. Our method produced profound ischemic changes at light microscopic level with its maximal effect elicited at 7 days and a considerable behavioral deficit with its peak at 4 days.

In previous studies, three phases were reported at the light microscopic level [4,6]. The first phase was reportedly happens in 0 h and 3 h groups and did in our study with only minimal axonal changes observed. Group 1 d in our study also showed slight edema, being mostly epineurial, but still no noticeable endoneurial changes. The second phase in our study was also in concordance with the aforementioned studies showing IFD and profound axonal edema at 7 days. Iida [4] also reported a third phase of fiber regeneration with only minimal edema and fiber debris at 28 and 42 days of reperfusion; but, as our study included groups up to 7 days, changes of third phase were not observed.

Regarding the behavioral score, our results were slightly different from the previous models. Iida et al. reported maximal behavioral deficit to happen at day 7 of reperfusion, while in our model this occurred at day 4 with a slight recovery at day 7. As Iida and his colleagues' study did not include a 4 day group, we can not make a judgment whether our model induces an earlier maximal deficiency. They used a 20 score scale to investigate behavioral performance and observed score 4 at 7 days (20% of the max) while in our study score 6 of an 11 score scale (54% of the max) was seen at 7 days. Although, inter-observer bias should be taken into consideration, it seems that our model failed to induce a neurologic deficiency so severe as the above-mentioned method. This could be explained by the extent of arteries cross clamped in their method. In spite of this difference, the simplicity of our modified method and its still-considerable induced functional defect in the limb make it rational to apply in experimental studies.

Briefly, our results show that common iliac artery and femoral artery clamping induces I/R injury in rat sciatic nerve. This method provides the obvious advantage of producing an easily induced moderate to severe behavioral deficit over the current methods and can be used in experimental studies designed to evaluate the effect of therapeutic candidates on I/R injury in peripheral nerves.

## Competing interests

The authors declare that they have no competing interests.

## Authors' contributions

MN participating in drafting the manuscript. RR participating in drafting the manuscript and statistical analysis. GF participating in statistical analysis and study design. MRR: Preparing the revised paper and drafting the manuscript. SMR participating in setting up the method.

ABF participating in setting up the method. FAA carried out pathologic assessment.

ARD participated in the design and cordination of the study. All authors read and approved the manuscript.
